# How and When Should Clinical Reasoning Be Taught in Undergraduate Medicine: A Systematic Review and Meta-Analyses

**DOI:** 10.5334/pme.1986

**Published:** 2025-12-29

**Authors:** Stuart Wark, Aaron Drovandi, Richard G. McGee, Faith O. Alele, Felista Mwangi, Bunmi Malau-Aduli, for the ACHIEVE network

**Affiliations:** 1 School of Rural Medicine, Faculty of Medicine & Health, University of New England, Armidale, New South Wales, Australia; 2 School of Medical Sciences, Faculty of Biology, Medicine and Health, University of Manchester, Manchester, Greater Manchester, United Kingdom; 3 John Hunter Children’s Hospital, Hunter New England Local Health District, Newcastle, New South Wales, Australia; 4 School of Medicine and Public Health, College of Health, Medicine and Wellbeing, University of Newcastle, Newcastle, New South Wales, Australia; 5 School of Health, University of the Sunshine Coast, Sunshine Coast, Queensland, Australia; 6 Academy for Collaborative Health Interprofessional Education and Vibrant Excellence (ACHIEVE), New South Wales, Australia

## Abstract

**Background:**

Clinical reasoning is a critical aspect of clinical practice, though there is considerable variation regarding how and when to teach this skill. This systematic review and meta-analyses examined the effectiveness of interventions that explicitly taught clinical reasoning in undergraduate medical education and the optimal timing for introducing interventions.

**Methods:**

A systematic (PRISMA 2020) search of the SCOPUS, MEDLINE, CINAHL, PsycINFO, ERIC, and Informit databases was conducted from 1 January 2014 to 31 December 2024. The quality of studies was assessed using the Quality Assessment with Diverse Studies tool. Pooled estimates and 95% confidence intervals (CIs) were estimated using both random and fixed effects meta-analyses.

**Results:**

The final sample included 50 studies, of which 46 (92%) reported a measurable improvement. Small-group teaching generally achieved better outcomes, with technology and serious game innovations further improving them. Meta-analysis of six randomised control trials using a random effects model showed an overall significant result (MD 2.23; 95% CI: 0.67, 3.80; I^2^ = 88%). A subgroup analysis indicated that interventions undertaken in pre-clinical years (MD 0.32; 95% CI: –3.99, 4.64; I^2^ = 88%) did not result in significant improvements, whereas interventions in the clinical years were significant (MD 1.89; 95% CI: 1.06, 2.72; I^2^ = 88%). A second subgroup analysis showed that interventions based on face-to-face workshops (MD 1.74; CI: 1.19, 2.28; I^2^ = 88%) were significant.

**Conclusions:**

The findings suggest that small-group activities, such as interactive online modules, may lay a foundation for early-year students, while skills-based workshops and serious games progressively refine and enhance clinical reasoning. Future research should focus on longitudinal outcomes and standardised assessment measures across diverse educational contexts.

## Introduction

Clinical reasoning is a concept that practicing clinicians often intuitively understand, yet it remains challenging to define precisely within educational environments [1]. Although clinical reasoning has been described in a variety of ways—sometimes with conflicting definitions—it is generally accepted as the iterative process of gathering and evaluating information from the patient and other sources to establish a diagnosis, predict a likely prognosis, and formulate an appropriate management plan [2]. Given its central role in patient care, clinical reasoning has long been regarded as one of the most critical components of medical and healthcare practice [3]. Despite its importance, the absence of a universally agreed-upon definition contributes to frequent conceptual misunderstandings of clinical reasoning in medical education. As a result, clinical reasoning is often poorly taught, or not taught explicitly at all, within undergraduate medical curricula [4]. Inconsistent and disparate approaches to the teaching of clinical reasoning skills across institutions risk leaving graduates inadequately prepared for clinical practice [5], which may ultimately contribute to avoidable diagnostic errors [6].

One complicating factor in developing clinical reasoning skills at the undergraduate level of medical education is the need to integrate two distinct spheres of knowledge. This process is best understood through the cognitive theory of illness scripts [7], which are structured mental frameworks of diseases that individuals develop to facilitate pattern recognition. Initially, students build the basic scaffolding for these scripts in classroom-based settings (script formation), with tutor-guided instruction on how to obtain and systematically analyse all potentially pertinent information. However, these foundational scripts are often generic and lack nuance. The second crucial element is clinical experience, which is essential for enhancing and enriching these scripts (script refinement). It is through healthcare-based clinical placement that students gain practical exposure to key conditions and learn how case-specific factors, such as comorbidities, social circumstances and atypical presentations, can shape how a disease manifests [8,9]. This practical exposure allows students to move beyond recognizing ‘classic’ presentations and begin to develop the sophisticated, flexible illness scripts that underpin expert clinical reasoning [7]. Illness scripts become more sophisticated and nuanced over time as the student gains experience, with the evolving cognitive framework assisting in efficiently identifying a disease and also incorporating new or contradictory information for different presentations of a certain condition. However, this perception of required sequencing—that prior exposure to a disease is necessary before clinical reasoning can be effectively learned—has contributed to uncertainty about the optimal timing of formal instruction [10]. As a result, there is no globally standardised approach across undergraduate medical programs, although it is generally acknowledged that simple clinical reasoning cases can be introduced in the foundational years before progressing to more complex presentations as students’ knowledge base increases in later years [8,9,10,11].

The aim of this systematic review was to evaluate the current evidence on the effectiveness of explicitly teaching clinical reasoning in undergraduate (pre-registration) medical education. Specifically, the review sought to identify the program year in which clinical reasoning teaching interventions were implemented and to determine whether these interventions were consistently embedded and scaffolded across all stages of the curriculum. While previous systematic reviews, such as that conducted by Xu et al. [12], have explored approaches to teaching clinical reasoning and identified that generally most interventions will see some level of improvement, the recent literature has not explicitly examined these models in relation to both the optimal timing for introducing such interventions and the methods used. Therefore, this review was designed to address these gaps by identifying the types of interventions that could be recommended for implementation in undergraduate medical programs and the most appropriate stage at which to introduce them.

## Methods

This systematic review was reported in line with the Preferred Reporting Items for Systematic Reviews and Meta-Analyses (PRISMA) guidelines [13].

### Search strategy

Six databases (SCOPUS, Medline, CINAHL, PsycINFO, ERIC, and Informit) were searched for studies on programs or courses that explicitly taught clinical reasoning to undergraduate medical students and explicitly described the outcomes of their interventions. The search string was developed by all authors and combined two term groups to interrogate the title, abstract and full-text fields in the databases. The two search term groups were:

Term Group 1- clinical judgement OR clinical judgements OR clinical reasoning OR judgement, clinical OR judgements, clinical OR reasoning, clinical OR clinical decision making OR decision-making, clinical OR decision-making, medical OR medical decision making OR diagnostic reasoning.

Term Group 2- education, medical, undergraduate OR education, undergraduate medical OR medical education, undergraduate OR undergraduate medical education.

The search strategy captured articles published across ten years from 1 January 2014 to 31 December 2024. All search results were imported into the online program Covidence [14] for screening.

### Study selection

Eligible articles were peer-reviewed publications written in English, published between 1 January 2014 and 31 December 2024. Studies were included if they involved undergraduate medical students and reported on the outcomes of educational interventions that explicitly taught clinical reasoning. Articles were excluded if they focused solely on the assessment of clinical reasoning skills without describing the associated teaching methods.

Two authors (SW and BMA) were involved in the screening process, with each independently screening studies in the online Covidence software. Any arising disagreements were resolved by consensus with the remaining authors. Reference and citation lists of all eligible studies and relevant systematic reviews were manually searched to identify any additional articles not initially identified by the nominated search strategy.

### Data extraction

Two authors (FM and BMA) conducted data extraction, and a third author (SW) independently reviewed the output. Standardised data extraction tables were developed and reviewed by all authors prior to commencement of data extraction. Data extracted from each of the included studies (as provided and/or as relevant) were: Article title; Author detail; Year of publication; Study Aim; Location; Study design; Study population; Data collection instrument/s; Intervention/s; Method of teaching; Method of assessment; Efficacy of intervention; Outcome measure/s; Study findings; and, Any additional information as relevant.

### Data synthesis and analysis

Quantitative data were reported as means and standard deviations (SD) or, where appropriate, as medians and interquartile ranges, while categorical data were presented as numbers and percentages. Qualitative data were reviewed and summarised by two authors (FM and BMA) in relation to the individual study findings. Meta-analyses of studies that employed a randomised controlled trial (RCT) design were performed by AD using RevMan^1^ software to assess the effectiveness of clinical reasoning teaching interventions. Means and SD from each RCT were extracted for inclusion in the meta-analyses. Both random-effects and fixed-effects models were initially applied to calculate pooled mean differences and 95% confidence intervals. Two subgroup meta-analyses—based on intervention type and year of commencement—were pre-specified to align with the key objectives of the review [15,16].

### Evaluation of quality of the reviewed studies

A methodological quality and bias assessment of the included articles was undertaken independently by two authors (FM and BMA) using the ‘Quality Assessment with Diverse studies’ (QuADS) tool. QuADS was designed to provide a unified framework for evaluating the quality of studies employing a range of research methodologies and has demonstrated good inter-rater reliability and validity [17,18]. The tool assesses 13 pre-defined criteria, each scored from 0 to 3, for a maximum possible score of 39 points [18]. Higher total scores indicate higher assessed methodological quality; however, no standard interpretation thresholds are prescribed for total scores. Prior to commencing the review, the authors developed and agreed upon a scoring model to categorise papers as ‘lower quality’ (<60%), ‘moderate quality’ (60–79%), or ‘high quality’ (≥80%). A lower quality rating was not meant to be automatically interpreted as identifying a fundamental flaw in the study, but rather as an indication of potential limitations and/or lack of effective reporting. It was agreed that studies assessed as lower quality would not be automatically excluded from the systematic review. Instead, they would be retained and their findings considered with appropriate caution. However, for the purposes of the meta-analyses, it was decided a priori that only studies assessed as moderate or high quality would be included.

## Results

**Figure 1:**
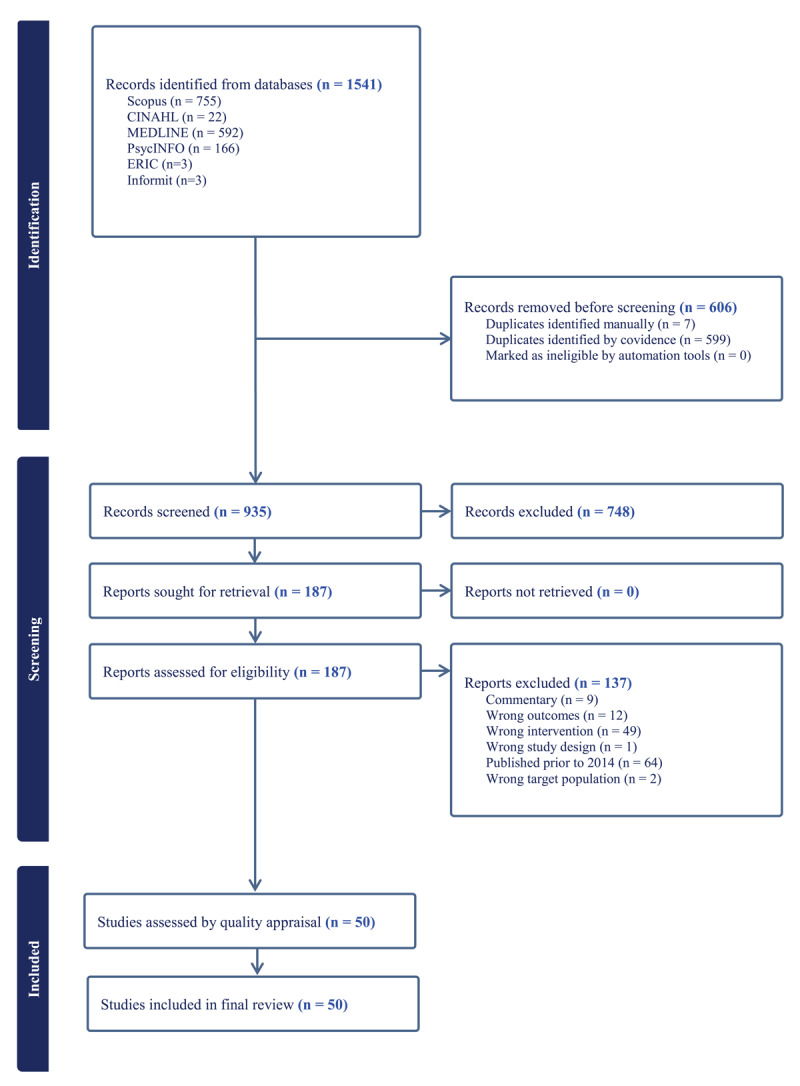
PRISMA FLOWCHART.

The initial database search identified a total of 1541 articles. Covidence software detected 606 duplicates, and the authors manually identified and removed an additional 7. Titles and abstract screening of the remaining 935 papers resulted in the exclusion of 748 articles based on the pre-determined inclusion and exclusion criteria. Full-text screening of the remaining 187 articles led to the removal of a further 137 articles. This process resulted in a final sample of 50 papers included in the review. The PRISMA flowchart for the selection process is presented in Figure 1.

### Study characteristics

Overall, half of the included studies were conducted in two countries, Germany (14) and the USA (11). Other countries represented in more than one article included the UK (3), the Netherlands (3), Canada (3), Hong Kong (2) and Brazil (2). There was considerable diversity in study designs, mainly encompassing quasi-experimental studies, variations on randomised controlled trials, and qualitative methodologies, amongst others. Approaches for evaluating the effectiveness of the clinical reasoning teaching interventions were equally varied and included pre- and post-test analyses, concept mapping and diagnostic performance assessments. The studies covered a broad range of medical sub-disciplines, from general medicine to specialty areas such as ophthalmology, surgery and dermatology. Due to the heterogeneity in study designs, intervention types, and participant populations, direct outcome comparisons across the full sample were limited. Nonetheless, only four studies [19,20,21,22] reported no measurable improvements following the described interventions. Some of the 46 papers that reported improvements used methodologies, such as qualitative approaches, that do not translate to measurable statistical significance.

Table 1 below presents a summary of the 50 papers categorised by year group, intervention type, country, and outcomes. For more details, please refer to the overview table of the key characteristics of all 50 studies in this review, which is included as a supplementary data file.

**Table 1:** Summary of included papers.


AUTHORS	STUDENT YEAR GROUP	INTERVENTION	COUNTRY	OUTCOMES

Augustin et al. [19]	Third	Clinical reasoning podcasts	USA	No statistical difference

Bonifacino et al. [23]	Third	Online modules and skills-based workshop	USA	Intervention showed superior performance

Braun et al. [20]	Fourth and Fifth	Problem representation	Germany	Diagnostic accuracy/efficiency did not differ

Brich et al. [24]	Third and Fourth	sTBL sessions and small group seminars	Germany	sTBL led to significantly better performance

Capaldi et al. [25]	Second	Clinical Integrative Puzzle (CIP)	USA	Significant for small group performance

Chadha et al. [26]	First	Virtual workshop on focused physical exam	USA	Improvement in mean scores (57% to 70%)

Chamberland et al. [27]	Third	Self-explanation with expert examples	Canada	Improvement over time for all groups

Chamberland et al. [28]	Third	Self-explanation with expert examples	Netherlands	Significant compared to control group

Cheng & Senathirajah [29]	Third and Fourth	Exposure to electronic health record	USA	Higher mean correct diagnoses

Chew et al. [30]	Final	Tutorial on cognitive biases with checklist	Malaysia	Significantly higher mean scores

Choi et al. [31]	Fourth	Training with reflection and feedback	South Korea	Significant better diagnostic accuracy

Costa Filho et al. [32]	Sixth	Reflection procedure on cases	Brazil	Increased diagnostic accuracy

Diemers et al. [33]	Third	Patient contact with laboratory work	Netherlands	Increased accuracy and explanations

Findyartini et al. [34]	Unspecified	PBL influenced by cultural perspectives	Australia and Indonesia	lower scores on the Flexibility in Thinking

Fukuta and Morgan [35]	Final	Video from a first-person perspective	UK	Improved performance in intervention group

Gilkes et al. [36]	Second	Diagnostic reasoning in clinical assessment	Australia	Improved diagnostic reasoning

Gouzi et al. [37]	Third	Interactive whiteboard (IWB)	France	Improved diagnostic skills

Hakim et al. [38]	Second	Real-life cases with audience response	United Kingdom	High consistency and correct decisions

Harendza et al. [39]	Final	Multi-unit course on uncertainty	Germany	Self-assessed improvement

Hayward et al. [40]	Second	Online virtual patient survey	Canada	Consistent diagnostic likelihood estimates

Hege et al. [41]	Unspecified	Concept mapping tool with virtual patient	Europe	Significant differences between maps

Houchens et al. [42]	Unspecified	Direct clinical teaching during rounds	USA	Successful teaching categorised in themes

Keemink et al. [43]	Second	Case-based clinical reasoning (CBCR)	Netherlands	Significant difference in illness script

Kelekar & Afonso [44]	Second	Whole case approach and self-explanation	USA	Higher scores on differential diagnosis

Kiesewetter et al. [21]	Third, Fourth and Fifth	Whole case approach and self-explanation	Germany	No significant differences

Kiesewetter et al. [45]	Third to Sixth	Virtual patients in clinical reasoning	Germany	High knowledge lead to better outcomes

Klein et al. [46]	Unspecified	Clinical case vignettes with instruction	Germany	Improvement in clinical reasoning

Kleinert et al. [47]	Third	Teaching module on operating procedures	Germany	Significant improvement

Koenemann et al. [48]	Unspecified	Clinical case discussions (CCD)	Germany	Significant increases in clinical reasoning skills

Lai et al. [49]	Fourth	Group history-taking with feedback	Taiwan	Training improved clinical reasoning skills.

Leung et al. [50]	Fifth	Virtual patient sessions on acute pain	Hong Kong	Case studies reinforced clinical learning

Lütgendorf-Caucig et al. [51]	Unspecified	Case-based exercises for clinical reasoning	Austria	Significant knowledge acquisition

Maciuba et al. [52]	First and Second	Case-based exercises for clinical reasoning	USA	Focus on problem list creation and diagnoses

Middeke et al. [53]	Final	Teaching sessions of either EMERGE or PBL	Germany	EMERGE group scored significantly higher

Middeke et al. [54]	Fifth	Computer-based Serious Game	Germany	Students scored significantly higher

Moghadami et al. [55]	Fourth	Teaching workshops using illness script	Iran	Improved students’ clinical reasoning skills

Mutter et al. [56]	Fourth	Patient case scenarios for chest pain sessions.	USA	Significant mean difference

Peacock & Grande [57]	First	Real patients presenting histories	USA	Improved clinical decision-making

Plackett et al. [58]	Final	eCREST, an online simulation tool,	UK	Improved ability to gather information

Raupach et al. [59]	Fourth and Fifth	Serious game simulation	Germany	Performance scores increased

Roberti et al. [60]	First to Fifth	Traditional medical education curriculum	Brazil	Difference between pre and clinical years.

Rosby et al. [61]	Second	Online chest X-ray cases training phase	Singapore	Significantly higher diagnostic accuracy

Rumayyan et al. [62]	Second	Instructional approach of signs/symptoms.	Saudi Arabia	Higher mean diagnostic accuracy

Schubach et al. [22]	Fourth and Fifth	Virtual patients with different approaches	Germany	No significant differences

Schuelper et al. [63]	Fourth	Computer-based seminars	Germany	Significant difference in retention scores

Scott et al. [64]	Final	Simulation teaching day	UK	Simulation supported textbook diagnosis

Weidenbusch et al. [65]	Unspecified	Live discussion and printed cases	Germany	Significant knowledge improvements

Wu et al. [66]	Third to Fifth	Online learning program	China	Significant improvement

Wu et al. [67]	Third and higher	Simulated cases of kidney disease	Hong Kong	Improved reasoning process

Zagury-Orly et al. [68]	First	Student-Generated Reasoning Tool (SGRT)	USA	Significantly higher scores


### Quality Assessment

The quality assessment results for the reviewed 50 studies are presented in Table 2, with papers listed alphabetically by lead author. Quality scores ranged from 44% to 95%. Of the 50 studies, 33 were assessed as being of high quality and 14 as moderate quality. Only three papers were classified as lower quality, with scores of 44% [29], 49% [35] and 51% [38]. The main areas where these lower-scoring studies [29,35,38] underperformed were in relation to the criteria of Analysis justified, Analysis appropriate, and Stakeholder considered. Following independent review and consensus discussion among the authors, it was agreed that these three studies would be retained in the systematic review, although their findings would be interpreted with caution in relation to their relevance and significance. Notably, none of these lower-quality studies reported on randomised controlled trials and thus did not require exclusion from the proposed meta-analyses.

**Table 2:** Quality assessment outcomes for the included studies (n = 50) conducted using the QuADS tool.


	STUDY (YEAR)	THEORY/CONCEPT	AIMS	SETTING	STUDY DESIGN	SAMPLING	DATA COLLECTION TOOLS CHOICE RATIONALE	TOOL FORMAT AND CONTENT	PROCEDURE DESCRIPTION	RECRUITMENT DATA	ANALYSIS JUSTIFIED	ANALYSIS APPROPRIATE	STAKEHOLDER CONSIDERED	STRENGTHS AND LIMITATIONS	TOTAL SCORE (%)

1.	Augustin et al. (2022) [19]	3	1	3	2	3	3	3	3	3	3	3	0	3	33 (85)

2.	Bonifacino et al. (2019) [23]	3	1	3	3	3	3	3	3	3	3	3	2	2	35 (90)

3.	Braun et al. (2019) [20]	2	1	2	3	3	2	3	3	2	3	3	2	2	31 (79)

4.	Brich et al. (2017) [24]	3	2	3	2	3	2	3	3	3	3	3	2	2	34 (87)

5.	Capaldi et al. (2015) [25]	3	3	3	3	3	3	3	3	3	3	3	2	2	37 (95)

6.	Chadha et al. (2021) [26]	1	1	3	3	3	3	3	3	3	0	2	2	2	29 (74)

7.	Chamberland et al. (2015a) [27]	2	2	3	3	2	2	3	3	3	3	3	2	2	33 (85)

8.	Chamberland et al. (2015b) [28]	2	2	3	3	2	2	3	3	3	3	3	2	1	32 (82)

9.	Cheng et al. (2022) [29]	2	2	1	3	2	1	3	2	1	0	0	0	0	17 (44)

10.	Chew et al. (2016) [30]	3	2	3	2	1	2	3	3	1	3	3	2	2	30 (77)

11.	Choi et al. (2020) [31]	2	2	3	3	3	1	3	3	3	3	3	2	2	33 (85)

12.	Costa Filho et al. (2019) [32]	2	3	3	3	2	3	2	3	3	3	3	0	2	32 (82)

13.	Diemers et al. (2015) [33]	1	3	2	2	2	0	3	3	3	2	3	0	3	27 (69)

14.	Findyartiniet al. (2016) [34]	3	2	3	3	3	3	3	3	1	3	3	2	2	34 (87)

15.	Fukuta and Morgan (2018) [35]	1	1	2	3	2	1	3	2	2	0	0	0	2	19 **(**49)

16.	Gilkes et al. (2022) [36]	2	2	3	3	3	3	3	3	2	2	3	2	3	34 (87)

17.	Gouzi et al. (2019) [37]	1	3	3	3	2	2	3	3	3	3	3	2	2	33 (85)

18.	Hakim et al. (2023) [38]	1	1	3	3	0	3	3	2	3	0	0	0	1	20 (51)

19.	Harendza et al. (2017) [39]	2	2	3	3	2	3	3	2	2	2	3	0	3	30 (77)

20.	Hayward et al. (2016) [40]	3	3	3	3	2	3	3	3	2	2	3	0	2	32 (82)

21.	Hege et al. (2018) [41]	3	3	3	3	2	3	3	3	3	3	3	1	2	35 (90)

22.	Houchens et al. (2017) [42]	2	3	3	3	3	3	3	3	3	2	3	0	3	34 (87)

23.	Keemink et al. (2018) [43]	2	3	3	3	2	3	3	3	3	3	3	2	2	35 (90)

24.	Kelekar and Alfonso (2020) [44]	2	2	2	2	2	0	2	2	2	3	3	1	2	25 (64)

25.	Kiesewetter et al. (2016) [21]	3	3	2	3	2	3	3	3	1	3	3	2	2	33 (85)

26.	Kiesewetter et al. (2020) [45]	2	3	2	3	3	3	3	3	2	3	3	2	2	34 (87)

27.	Klein et al. (2019) [46]	2	3	2	3	3	3	3	3	3	3	3	2	2	35 (90)

28.	Kleinert et al. (2015) [47]	1	2	3	3	2	3	3	3	2	2	3	2	1	30 (77)

29.	Koenemann et al. (2020) [48]	1	3	3	3	2	2	2	3	2	0	0	2	2	25 (64)

30.	Lai et al. (2022) [49]	1	3	3	3	2	3	3	3	1	3	3	2	2	32 (82)

31.	Leung et al. (2015) [50]	2	2	3	3	2	3	3	3	2	3	3	2	2	33 (85)

32.	Lütgendorf-Caucig et al. (2017) [51]	2	1	3	3	2	3	3	3	1	3	3	2	1	30 (77)

33.	Maciuba et al. (2023) [52]	3	3	3	3	2	3	3	2	2	2	3	0	3	32 (82)

34.	Middeke et al. (2018) [53]	2	2	3	3	2	3	3	3	3	3	3	2	3	35 (90)

35.	Middeke et al. (2020) [54]	1	3	3	3	2	3	3	3	3	3	3	2	3	35 (90)

36.	Moghadami et al. (2021) [55]	3	2	3	3	3	2	2	3	2	2	3	3	3	34 (87)

37.	Mutter et al. (2020) [56]	3	3	3	3	2	3	3	3	3	3	3	2	2	36 (92)

38.	Peacock et al. (2015) [57]	1	2	3	3	2	3	3	3	1	2	3	0	2	28 (72)

39.	Plackett et al. (2020) [58]	2	2	2	3	3	3	3	3	3	3	3	2	2	34 (87)

40.	Raupach et al. (2021) [59]	2	3	3	3	2	3	3	3	3	3	3	2	3	36 (92)

41.	Roberti et al. (2016) [60]	2	2	3	3	2	2	3	2	2	3	3	0	2	29 (74)

42.	Rosby et al. (2018) [61]	3	2	3	3	2	2	3	3	1	3	3	0	2	30 (77)

43.	Rumayyan et al. (2018) [62]	2	3	3	3	2	1	3	3	3	3	3	2	2	33 (85)

44.	Schubach et al. (2017) [22]	2	2	3	3	3	3	3	3	3	3	3	2	2	35 (90)

45.	Schuelper et al. (2019) [63]	2	2	3	3	2	2	3	3	3	3	3	2	3	34 (87)

46.	Scott et al. (2020) [64]	1	2	3	3	3	3	3	2	3	3	3	2	2	33 (85)

47.	Weidenbusch et al. (2019) [65]	3	3	3	3	3	3	3	3	2	3	3	2	2	36 (92)

48.	Wu et al. (2014) [66]	2	3	2	3	2	3	3	3	2	0	3	2	2	30 (77)

49.	Wu et al. (2016) [67]	2	3	1	3	2	3	3	3	2	3	3	2	2	32 (82)

50.	Zagury-Orly et al. (2022) [68]	3	1	3	3	2	3	3	3	2	3	3	2	2	33 (85)


### Year and mode of delivery of clinical reasoning teaching intervention

For analysis, papers were stratified into categories based on whether their sample population included students at: first year; second year; third year; fourth year; final years (fifth or sixth year); or across multiple years. It is acknowledged that some medical programs offer a four-year degree, and therefore their ‘final years’ cohort would technically be fourth-year students. However, a brief but admittedly non-comprehensive review of medical programs worldwide indicated that such courses were predominantly offered at the postgraduate level and therefore were not eligible for inclusion in this review.

Overall, three studies examined the impact of interventions specifically for first-year students [26,57,68], with eight, seven and five, respectively, for second [25,36,38,40,41,44,61,62], third [19,24,27,28,33,37,47], and fourth [32,49,55,56,63] year students. Final-year (including fifth- and/or sixth-year) students were the most represented year group, being the sole focus of nine studies [30,32,35,39,50,53,54,58,64]. Thirteen of the included papers described studies with samples derived from students across multiple year groups [20,21,22,24,29,34,45,48,52,59,60,66,67]. It was not possible to accurately determine the year group for five [41,42,46,51,65] of the studies.

### First-year student cohorts

Only three studies [26,57,68] described interventions that specifically targeted students in the first year of their medical education, of which one was a pilot study [57]. All three studies reported some improvement in participants’ clinical reasoning skills following the respective interventions, albeit not necessarily at a level of statistical significance, and none were randomised controlled trials and were therefore not included in the meta-analyses. Chadha et al. [26] implemented a virtual workshop aimed at developing clinical reasoning skills and enhancing the generation of differential diagnoses among first-year medical students. Using a pre- and post-test design, they demonstrated significant improvements in student performance, with overall positive feedback following the workshop. These results indicate that even brief teaching interventions early in medical training can potentially lead to meaningful improvements in clinical reasoning skills/abilities. Similarly, Zagury-Orly et al. [68] reported positive outcomes following the implementation of a Student-Generated Reasoning Tool (SGRT) during small group activities. This tool guided students through a structured hypothesis-generation framework, encouraging systematic evaluation of clinically relevant information. Students who engaged with the SGRT were reported to be 5 times more likely to accurately answer pathophysiology questions than a control group. However, the study also found that response accuracy was significantly lower when students were assessed individually rather than in groups.

A pilot study by Peacock & Grande [57] further supported the notion that implementing interventions in the first year of medical education can enhance the development of clinical reasoning skills. Their study incorporated online assignments into the first-year pathology curriculum, in conjunction with exposure to real patients with actual health conditions, to strengthen clinical reasoning skills. The intervention improved clinical decision-making within the cohort, as demonstrated by enhanced differential diagnosis formulation. Additionally, the authors noted that engaging with real patients facilitated students’ ability to better apply relevant clinical context to their basic science knowledge.

### Second year student cohorts

Eight studies [25,36,38,40,43,44,61,62] implemented interventions targeting second-year medical students, all of which reported at least some positive outcomes in improving clinical reasoning skills. Kelekar & Afonso [44] implemented a new clinical reasoning curriculum, which resulted in students achieving higher scores in the assessment of differential diagnoses. Similarly, Gilkes et al. [36] reported improvements following the development of a targeted training package designed to reduce diagnostic reasoning errors based on examiner observations during clinical assessment activities. They noted that the training improved the teaching of clinical reasoning, which, in turn, led to reductions in both the types and rates of diagnostic errors made by students. Keemink et al. [43] developed a case-based clinical reasoning course, which showed improved diagnostic performance, while Rosby et al. [61] also reported significantly faster and more accurate diagnostic responses following online training. Although assessed as lower quality in the QuADS evaluation, Hakim et al. [38] also reported comparable findings in their integrated teaching session, which combined respiratory physiology with clinical reasoning, and reported improved student understanding of clinical reasoning concepts.

Capaldi et al. [25] described the development of the Clinical Integrative Puzzle (CIP) as a tool for teaching and evaluating clinical reasoning in small group settings. Their study reported high feasibility, as indicated by the time required for CIP completion, and acceptable reliability measured by odd–even item reliability analysis. However, they found only modest evidence supporting the tool’s validity. In a novel approach, Hayward et al. [40] utilised an online case-based learning tool grounded in script theory. While participants reported the tool to be a valuable learning experience, no directly comparable performance data against other student cohorts was available. Rumayyan et al. [62] compared two instructional approaches for teaching diagnostic reasoning: the self-explanation method, where students provided pathophysiological explanations for clinical findings, and the hypothetico-deductive method, where students sequentially hypothesised possible diagnoses based on presented clinical signs and symptoms. Their findings indicated that students in the hypothetico-deductive group demonstrated superior overall diagnostic accuracy compared to those in the self-explanation group.

### Third-year student cohorts

Seven studies [19,23,27,28,33,37,47] focused on cohorts composed of third-year medical students, and they employed a wide variety of approaches to teaching clinical reasoning. Augustin et al. [19] investigated adding clinical reasoning podcasts to the standard curriculum but found no significant improvement in clinical reasoning performance in the intervention group compared with the control group. In contrast, there were five studies, including a pseudo-randomised control study by Bonifacino et al. [23] and quasi-experimental studies by Chamberland et al. [27], Chamberland et al. [28], Diemers et al. [33] and Kleinert et al. [47], that all reported positive outcomes by demonstrating improvements in clinical reasoning skills. The study by Gouzi et al. [37] took a slightly different approach, evaluating the feasibility of incorporating interactive whiteboards into clinical reasoning sessions to enhance students’ diagnostic reasoning. Their findings indicated improvements in the selection of appropriate diagnostic tests and in the interpretability of test results, and a reduction in unnecessary test ordering among students in the whiteboard intervention group compared to the control group. However, as both groups received general clinical reasoning teaching, it was not possible to determine the overall effectiveness of the intervention.

### Fourth-year student cohorts

Most of the five studies [31,49,55,56,63] that tested interventions with fourth-year medical students focused on a more nuanced approach to clinical reasoning, such as through the lens of sub-specialities or acute illness. For example, Choi et al. [31] examined the use of reflection and expert feedback in teaching clinical reasoning within dermatology. Outcomes of the intervention, which included reflection and feedback, were compared with those of the control groups receiving either additional lectures or the standard outpatient clinical placement. Their results indicated that the reflection and feedback group achieved significantly greater diagnostic accuracy in evaluating dermatologic conditions than the two control groups.

Mutter et al. [56] examined whether the use of manikins could enhance clinical reasoning skills in acute illness scenarios. Students were assigned to work through a facilitated chest pain case scenario either with or without a manikin. The manikin group achieved significantly higher test scores. The study by Lai et al. [49] focused on teaching clinical reasoning through group history-taking with immediate feedback from a facilitator. Students showed improved clinical reasoning skills, demonstrated by better identification of key clinical information and enhanced diagnostic accuracy. Similarly, Moghadami et al. [55] investigated the use of teaching workshops based on the illness script method to improve clinical reasoning skills. Their results showed that students in the intervention group achieved significantly higher post-test scores on a script-concordance test than those in the control group.

Schuelper et al. [63] compared two different methods of delivering clinical reasoning instruction: repeated exposure to video-based versus text-based ‘feature questions’. Students participated in weekly seminars containing key feature cases and self-nominated to receive the material in either video or text format. Participants completed assessments at entry, exit, and a later retention exam. While no significant differences were observed between the groups in entry or exit scores, students with greater exposure to video-based materials performed significantly better on the retention test compared to those who predominantly engaged with text-based cases.

### Final year (fifth and/or sixth) student cohorts

Nine studies [30,32,35,39,50,53,54,58,64] focused specifically on interventions targeting students in the final year of their medical programs, most of which examined more advanced concepts inherent to clinical reasoning. Harendza et al. [39] developed and implemented a bespoke clinical reasoning course that actively incorporated the concept of managing ‘uncertainty’ within complex patient scenarios. The findings reported improvements in diagnostic performance and the adoption of more sophisticated approaches to clinical case presentations. Similarly, Scott et al. [64] explored the teaching of clinical reasoning in the context of complex cases and diagnostic uncertainty. Their teaching model featured structured, facilitated debriefing sessions following simulation exercises. Unlike many of the other studies in this review, Scott et al. [64] highlighted specific areas where students encountered significant difficulties. Participants frequently became ‘disheartened’ when they were unable to establish a clear diagnosis, struggled to recognise when to seek guidance from the facilitators, and exhibited fixation on finding a definitive solution. The authors suggested that simulation experiences fostered unrealistic expectations of ‘textbook’ patient presentations, thereby reducing students’ cognitive flexibility. This rigidity in thinking hindered their ability to adapt to atypical or ambiguous clinical scenarios, leading to diagnostic inaction and uncertainty about how to proceed when a straightforward diagnosis was not evident.

Other studies considered the use of specialist tools to support the development and accuracy of clinical reasoning and decision-making. Chew et al. [30] evaluated the use of a specialist checklist introduced during tutorials and found that students using the checklist achieved significantly higher clinical decision-making scores than those in a control group. Middeke et al. [53] and Middeke et al. [54] investigated the use of serious games to support the development of clinical reasoning. In the first study [53], students who participated in EMERGE, an emergency ward simulation game, outperformed those in traditional PBL tutorials on key feature examinations. Their second study [54] showed that repeated exposure to virtual patient cases through EMERGE improved clinical reasoning for two specific case scenarios, although no significant differences were observed in other cases. Plackett et al. [58] reported that the use of eCREST (electronic Clinical Reasoning Educational Simulation Tool) improved students’ ability to accurately gather essential patient information, with students providing positive feedback on the model. Fukuta and Morgan [35], acknowledging that their study was rated as potentially lower quality, found that all students exposed to a first-person perspective video correctly diagnosed the condition, compared with 70% in the control group.

The remaining studies considered the teaching of clinical reasoning within defined sub-specialities. Costa Filho et al. [32] evaluated the impact of deliberate reflection on clinical reasoning in dermatology and found that students in the reflection group demonstrated higher diagnostic accuracy compared to the control group (49.7% vs 38.4%). Leung et al. [50] explored clinical reasoning in anaesthesia, using virtual simulated patient cases focused on acute pain management. They reported that formative assessment case studies helped reinforce the development of clinical reasoning skills in this context.

### Multiple year groups

Thirteen studies [20,21,22,24,29,34,45,48,52,59,60,66,67] included students from across multiple year levels, though most interventions were single, stand-alone activities rather than longitudinal, scaffolded programs. Roberti et al. [60] reported that early-year students relied mainly on basic science knowledge for clinical reasoning, before transitioning to pattern recognition as they progressed. Two studies [20,22] found no significant improvement following their interventions, highlighting challenges such as diagnostic skill gaps and premature closure. Braun et al. [20] provided structured scaffolding for reflection, with or without feedback, but observed no improvement in diagnostic accuracy or efficiency compared to a control group. The study identified contributing factors to diagnostic errors, including a lack of diagnostic skills, limited knowledge, and premature closure, highlighting areas for future focus. Similarly, Schubach et al. [22] investigated the use of podcasts as an intervention to support clinical reasoning but found no significant differences between the intervention and control groups.

In contrast, a general trend across the remaining studies was that the interventions designed to explicitly teach clinical reasoning led to measurable improvements in assessed clinical reasoning skills. For example, Koenemann et al. [48] implemented a peer-led clinical case discussion model, facilitated by a physician, to develop clinical reasoning skills in an elective unit open to students from any year group. They reported significant increases in the participants’ clinical reasoning skills at the end of the elective unit when compared to commencement. A similar approach was undertaken by Brich et al. [24], who compared small-team-based learning (sTBL) with small-group interactive seminars for the development of neurological clinical reasoning skills in third- and fourth-year students. They found that sTBL improved clinical reasoning assessment in certain topic areas, although it was not consistently superior across all domains. Raupach et al. [59] adopted a serious game approach to teaching clinical reasoning. They simulated an emergency department environment with students exposed to virtual patient cases. They reported improvements in clinical reasoning skills within the exposed group over time and compared with the control group.

### Not stated (Unspecified) year groups

Five papers [41,42,46,51,65] did not provide sufficient information to clearly determine the year level of the student cohorts targeted by the interventions. Therefore, these papers were reviewed more generally in relation to their overall findings on the teaching of clinical reasoning to medical students. Among these, three studies were particularly relevant in providing broader context to the aims of this review. Houchens et al. [42] used an exploratory qualitative study design to evaluate clinical reasoning teaching to undergraduate students and to identify the techniques used by experts to teach and enhance clinical reasoning. Through direct observations of clinical reasoning teaching during hospital rounds, they identified four key strategies employed by experts:

1. emphasising the organisation and prioritisation of pertinent information;
2. highlighting important underpinning knowledge;
3. encouraging conscious thinking (thinking aloud); and
4. connecting reasoning processes to the existing literature.

Klein et al. [46] examined different instructional approaches for developing clinical reasoning. In this study, students were tasked with reporting on clinical cases and were then guided through an analysis of the errors they had made. The authors found that the ‘learning from errors’ model significantly improved clinical reasoning performance, although providing additional prompts did not offer further benefits.

Weidenbusch et al. [65] investigated the impact of different presentation formats for clinical case discussions on the development of clinical reasoning skills. They compared outcomes across three groups: live in-person discussion training; video recordings of live discussions; and provision of printed case materials. Their findings showed significant improvements in the post-test scores for both the live discussion and video recording groups compared to the printed material group. However, no significant difference was observed between the live discussion and video recording groups in a delayed post-test.

### Meta-analyses of the RCTs to identify significant improvements

**Figure 2:**
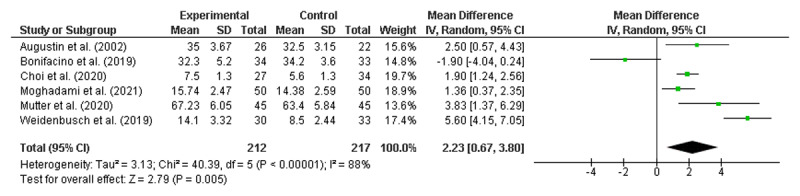
Overall result random effects model.

**Figure 3:**
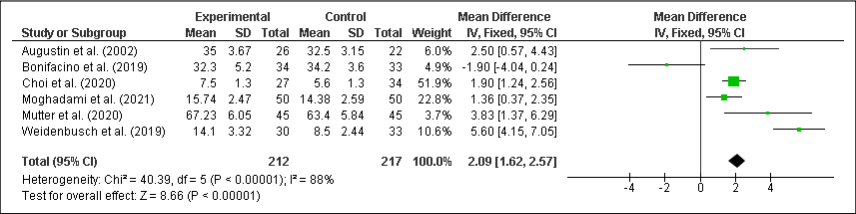
Overall result fixed effects model.

Meta-analyses were conducted using six randomised control trials [19,23,31,55,56,65]. As noted earlier, none of the studies identified as being ‘lower quality’ reported on randomised controlled trials and therefore did not require exclusion. All numerical data were reported as mean and standard deviation (SD). Given that 46 of the 50 included studies reported at least some positive findings, there was concern about publication bias, which would support the use of a fixed-effects model [69]. However, as there was also high potential for heterogeneity across the diverse range of interventions, meta-analyses were performed using both random effects (RE) and fixed effects (FE) [70], with the results from both models presented in Figures 2 and 3, respectively.

Both the RE and FE models demonstrated that clinical reasoning interventions were associated with improved student outcomes, with a mean difference of 2.23 (95% CI: 0.67, 3.80) in the RE model and 2.09 (95% CI: 1.62, 2.57) in the FE model. A high level of heterogeneity was noted (I^2^ = 88%).

**Figure 4:**
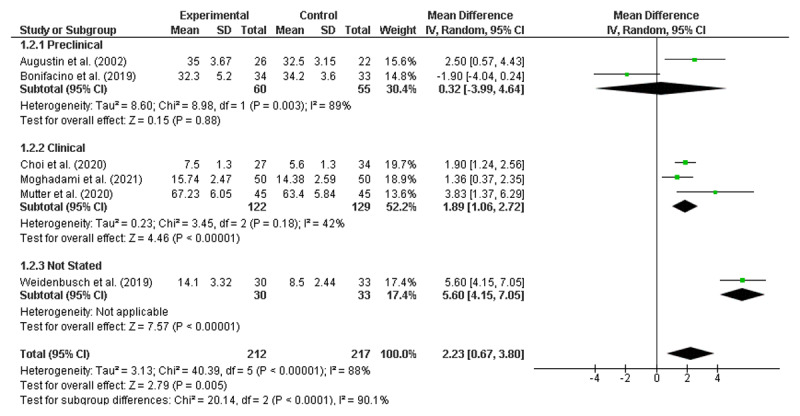
Pre-clinical, Clinical and Not-stated Intervention commencement (random effects).

**Figure 5:**
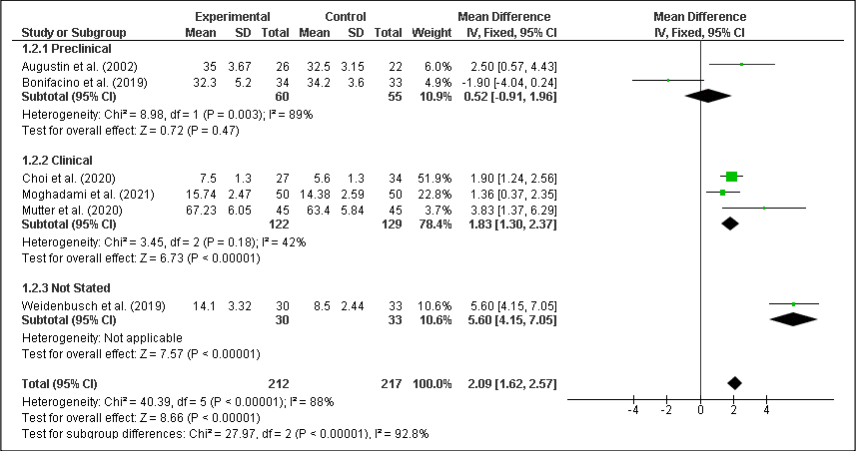
Pre-clinical, Clinical and Not-stated Intervention commencement (fixed effects).

Pre-specified subgroup analyses were also conducted using both RE and FE models [70]. Due to the limited number of studies, stratification by individual year level was not possible. Instead, studies were grouped by stage of training defined as Pre-clinical (Years 1–2); Clinical (Years 3–6); and Not Stated (see Figures 4 and 5). It is worth noting that no papers were included that focused solely on a Year 1 cohort.

Subgroup analyses by stage of training revealed significant improvements for interventions introduced during the clinical years with both models (RE: MD = 1.89; CI 1.06, 2.72; FE: MD = 1.83; CI 1.30, 2.37) but not for those introduced during the pre-clinical years (RE: MD = 0.32; CI –3.99, 4.64; FE: MD = 0.52; CI –0.91, 1.96). There was one paper in the subgroup analysis where the year of training was not stated (MD = 5.6; 4.15, 7.05).

**Figure 6:**
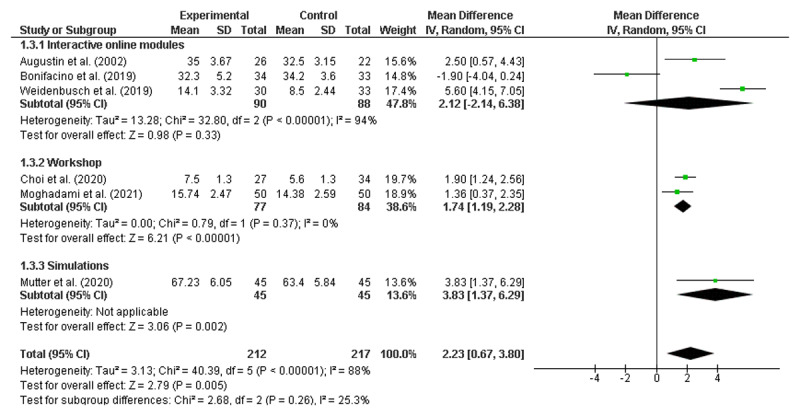
Interactive online, Workshop and Simulation Interventions (random effects).

**Figure 7:**
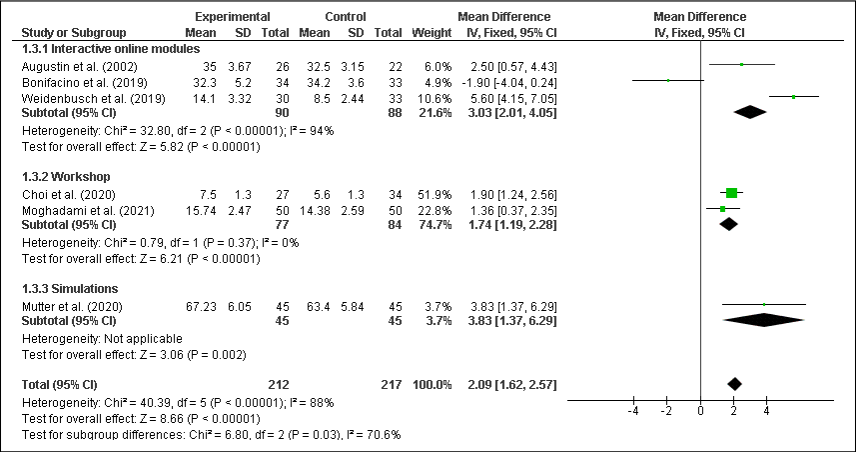
Interactive online, Workshop and Simulation Interventions (fixed effects).

A second subgroup analysis was performed by type of intervention (Interactive online, Workshop, or Simulation), as presented below in Figures 6 and 7, again using both RE and FE models.

Analysis by intervention type showed significant improvements for online interactive modules with the FE model (MD = 3.03; CI 2.01, 4.05) but not in the RE model (MD = 2.12, CI –2.14, 6.38). Face-to-face workshops indicated significant improvement in both the RE (MD = 1.74; CI 1.19, 2.28) and FE models (MD = 1.74; CI 1.19, 2.28). The simulation-based intervention analysis was one paper (MD = 3.83; CI 1.37, 6.29).

## Discussion

This systematic review and meta-analyses aimed to examine the current evidence regarding the effectiveness of explicitly evaluated clinical reasoning teaching interventions in undergraduate (pre-registration) medical education. As noted in the Introduction, it was thought likely that most interventions would see some level of quantifiable improvement. Therefore, this review also aimed to define the optimal stage within the medical program for introducing clinical reasoning instruction and to identify which types of interventions could be recommended for future implementation. Generally, the findings confirm that structured, active, and feedback-driven interventions can significantly enhance medical students’ clinical reasoning skills. Most (92%) of the studies reported that the described intervention reported some level of improvement, with the meta-analysis of six randomised control trials showing some overall significant improvements in student performance. The most notable improvements occurred when interventions were introduced during the clinical years of training.

### Timing of clinical reasoning interventions

Most of the included studies in this review focused on students in their clinical years (Years 3 and beyond). Sub-group analysis by training stage revealed that clinical reasoning interventions introduced during the clinical years were significantly effective, whereas those introduced during the pre-clinical years (Years 1–2) were not. Studies introducing interventions in the clinical years [e.g. 31,56] demonstrated improvements in diagnostic accuracy, problem-solving ability, and decision-making skills. In contrast, while some studies targeting first-year students [e.g. 26,57] showed early gains in basic reasoning processes, the magnitude and sustainability of these improvements appeared limited without ongoing reinforcement. This finding aligns with cognitive learning theories suggesting that successful teaching of clinical reasoning requires a critical mass of biomedical knowledge, evolved understanding and development of illness scripts, and clinical exposure to be applied meaningfully [73,74,75]. However, there was a lack of longitudinal multi-year studies that actively considered this concept. Generally, students in later years were seen to have accumulated sufficient foundational knowledge and clinical exposure to meaningfully engage with more complex reasoning processes. Interventions introduced in earlier pre-clinical years were less consistently effective, potentially reflecting the challenge of fostering sophisticated diagnostic reasoning in students who have not yet developed a broad knowledge base or clinical context.

Only three of the fifty studies specifically focused on Year 1 cohorts, which is where it may seem logical to introduce clinical reasoning, as it would have the greatest long-term impact on student learning. The subgroup meta-analysis indicated that clinical teaching was effective in the later years, but not in Years 1 and 2. This finding supports the argument that medical students need to have achieved a base level of both biomedical knowledge and sufficient clinical exposure before a genuine understanding of the reasoning behind decision-making is viable. On face value, this finding would endorse the concept of starting clinical reasoning after first year, but it is worth noting that all three of the studies [26,57,68] that used a first-year cohort reported improvements in students’ clinical reasoning skills, albeit not to an overall significant level.

The exclusion of year one students is hypothesised to be based on the presumption that the initial concepts of clinical reasoning cannot be taught concurrently with foundational knowledge and patient exposure sessions; however, it is possible that the simultaneous approach establishes an appropriate frame to guide student thinking as they further develop and deepen their understanding in future years. A study that highlighted problems associated with inflexibility in thinking among final-year students [64] would also seem to support the idea of introducing clinical reasoning in earlier years. These findings suggest a spiral curriculum approach [5] would be worth considering: early low-stakes exposure during pre-clinical years to introduce clinical reasoning frameworks, followed by more intensive, application-focused interventions during clinical training.

Following on from the concept of a spiral curriculum, it is worth noting that most studies did not report on programs that provided ongoing structured teaching of clinical reasoning skills across multiple years and instead mostly focused on one-off interventions of shorter duration. This is exemplified by the fact that many studies did not appear to identify the desired outcomes for graduates. It would seem logical that if the goal is to explicitly teach clinical reasoning to undergraduate medical students, and to teach it well, such teaching needs to be embedded across multiple year groups and should deliberately scaffold the teaching of skills to incorporate increasing knowledge. What is lacking in this group of studies is strong evidence on the effectiveness of clinical reasoning teaching designed specifically to build skills longitudinally across an entire program of study with the same cohort.

As noted in the Introduction, a point of consideration was to examine how the cognitive theory of illness scripts [7] was scaffolded across the teaching of clinical reasoning, and specifically whether programs built from script formation principles in the pre-clinical years into script refinement as the students gain exposure to a range of both patients and settings in the later years. The main factor that limits significant discussion on this issue relates to the lack of longitudinal or multi-year student studies that support investigation of whether the approach of using simple clinical reasoning cases in the foundational years, before progressing to more complicated presentations as the students’ knowledge base increases in later years [8,9,10,11], is appropriate. While 13 [20,21,22,24,29,34,45,48,52,59,60,66,67] of the included papers described studies across multiple year groups, Roberti et al. [60] was the only one to actively compare pre-clinical versus clinical student cohorts, noting that early-year students relied mainly on basic science knowledge for clinical reasoning before refining their illness scripts as they progressed. A common approach in many of these multi-year student projects was to provide the same intervention without differentiation or consideration of the participants’ underlying levels of knowledge or expertise, which should naturally vary depending on how many years of study the participants may have completed. It is therefore hard to make any genuine observations or recommendations based on the existing literature in this regard.

A brief review of medical curricula around the world would indicate that it is highly likely that most medical schools do teach clinical reasoning, either explicitly or implicitly, across their undergraduate program; what appears to be lacking from a medical education research perspective are studies that examine the longitudinal outcomes across year groups with respect to illness script development, but also which evaluate the duration of impact with respect to long-term outcomes for graduates. This is a clear recommendation for future researchers to consider.

### Effectiveness of clinical reasoning interventions

Across the 50 included studies, there was substantial diversity in teaching approaches, assessment modalities, and medical sub-specialty contexts. Intervention types ranged from interactive online modules, workshops, and serious games to virtual patient simulations, self-explanation techniques, and structured reflection activities. Despite this variability, a general trend emerged: interventions that explicitly targeted clinical reasoning development typically led to improvements in student performance across a range of outcome measures, including diagnostic accuracy, knowledge organisation, and information prioritisation. The meta-analysis of six papers supported these findings, showing that clinical reasoning interventions significantly improved student outcomes. Both random effects and fixed effects models confirmed the effectiveness of explicit clinical reasoning instruction and reinforced previous findings that clinical reasoning is amenable to targeted educational strategies [71,72].

A subgroup analysis categorised by intervention type indicated that face-to-face workshops produced significant improvements. Combined with the findings from the papers not included in the meta-analysis, this supports the idea that multiple delivery formats in small-group settings can successfully enhance clinical reasoning when appropriately designed and implemented. Interactive online modules were significantly effective using a fixed effects model, but this significance disappeared in the random effects model, and further investigation of such approaches is required. Across all the papers, effective interventions included interactive online modules [23,50], skills-based workshops [26,55], virtual patient simulations [31,54], self-explanation techniques [27], and serious games [59]. Importantly, interventions that incorporated structured feedback and deliberate practice produced superior outcomes compared to passive learning approaches, consistent with educational theory emphasising active engagement and immediate reinforcement [73]. In contrast, more passive interventions, such as clinical reasoning podcasts without structured reflection or feedback [19,22], were less effective, highlighting the importance of interactive learning environments.

However, these findings were not universal, and the use of podcasts as a tool did not appear to provide significant improvements. It is possible that the singular nature of this approach (i.e. students listening in isolation rather than in small groups) may have been another factor leading to this outcome. On initial consideration, these overall findings suggest that direct clinical reasoning interventions in small-group settings are likely to be beneficial for undergraduate medical students’ learning. However, it also supports the premise that there is still considerable divergence in whether clinical reasoning is explicitly taught and that this inconsistency in approach may be failing to adequately prepare graduates for entry into clinical practice [5].

## Practical Implications

These findings have significant practical implications for medical education curriculum design. Explicit teaching of clinical reasoning should be embedded systematically through a planned longitudinal approach across the medical program [79] via a spiralled curriculum [5], rather than relying on incidental development through clinical experience [71,76,77]. Early introductory sessions through low-stakes exercises in Years 1–2 can scaffold core thinking skills, such as hypothesis generation and information prioritisation [57,68] and prepare students to more effectively engage in deeper clinical reasoning once clinical placements begin. Additionally, formal structured clinical reasoning interventions should be embedded from the start of the clinical years, ideally integrating interactive simulations and online modules with increasing complexity and real-world fidelity as students progress [72,78]. Furthermore, blended teaching approaches that combine face-to-face workshops, online platforms, serious games, and small-group case discussions are likely to be the most effective and feasible across diverse institutional contexts, offering both scalability and interactivity. Finally, educators must recognise and directly address the challenges of uncertainty and diagnostic flexibility, especially in the later years, to prepare students for real-world clinical ambiguity. They must explicitly teach management of diagnostic uncertainty, flexibility in thinking, and reflective practice [39,64]. Future studies should focus on longitudinal tracking of clinical reasoning development over the course of medical training and explore how newer technologies (e.g. virtual reality, AI-based simulations) could be incorporated into clinical reasoning training.

## Strengths and Limitations

This paper presents a novel contribution to the literature base by providing a review of the effectiveness of teaching clinical reasoning interventions in undergraduate medical education. A particular strength of this article is the focus on identifying the year cohort in which specific interventions were implemented. The arising information provides guidance for medical educators in relation to both what interventions are effective and when to consider introducing a specific intervention into the curriculum.

While the meta-analyses assisted to further clarify what types of interventions are effective and when they should commence, caution is required in interpreting these results. It is acknowledged that the meta-analyses in this review exhibited high heterogeneity, included studies with small sample sizes, and had wide confidence intervals. There were also different outcome measures used across the papers included in the meta-analysis. These factors all indicate a lack of clarity around the actual effect. It has been argued for many decades that meta-analyses with small sample sizes are more likely to overestimate the interventions’ effect and that larger trials are less susceptible to publication bias [80]. However, in the context of undergraduate medical education settings with limited potential participants, randomised controlled trials in this area are highly unlikely to feature large participant numbers. Together, these concerns underline the need for additional research in this area in order to increase the precision of findings.

The review did not identify a large number of papers that commenced interventions in the first year of undergraduate medical training, or which reported findings across sequential years of study. It is considered possible that one of the reasons the authors did not find any such papers is due to the fact that the review specifically searched for projects with evaluation data. Research that reports on longer-term outcomes, such as that arising from interventions over many years, is rarer than papers which report one-off findings that are contained within a single year group.

Finally, half of the 50 included studies came from just two countries: Germany (14) and the United States (11). This bias suggests that readers should carefully consider the relevance of the overall findings to their own setting. This is particularly pertinent for regions where the delivery of medical education may differ substantially from that in Germany and the United States. Further, there was a lack of research from low- and lower-middle-income countries, and particular caution is required for those locations in relation to the generalisability of the findings.

## Conclusion

This systematic review and meta-analyses provide strong evidence that clinical reasoning can and should be explicitly taught in undergraduate medical education. Active, feedback-driven interventions introduced during the clinical years are particularly effective, while early pre-clinical exposure can help scaffold reasoning skills for later development. Structured, interactive approaches—tailored to the learner’s stage of knowledge and clinical experience—should form the foundation of clinical reasoning curricula. Moving forward, curriculum designers must invest in comprehensive, evidence-based strategies to equip future clinicians with the diagnostic acumen required for safe and effective practice.

## Data Accessibility Statement

All data generated for this review and meta-analysis is referred to within the main body of the document.

## Additional File

The additional file for this article can be found as follows:

10.5334/pme.1986.s1 Supplementary Data File. Summary of Study Characteristics.

## Footnotes

https://training.cochrane.org/online-learning/core-software#RevMan.
