# Supplementary material for: How and When Should Clinical Reasoning Be Taught in Undergraduate Medicine: A Systematic Review and Meta-Analyses
Source: Perspect Med Educ. 2025 Dec 29;14(1):1021–42. doi: 10.5334/pme.1986 (PMC12758103; doi:10.5334/pme.1986)
Supplement: Supplementary Data File. — Summary of Study Characteristics. [file pme-14-1-1986-s1.pdf]

Supplementary Data: Summary of Study Characteristics

Authors and Year of Publication	Location	Study Design/type	Study Population	Data Collection (Instrument)	How Clinical Reasoning Was Taught (Intervention)	How Clinical Reasoning Was Assessed	The Efficacy of the Intervention	Outcome Measure	Study Findings
Augustin et al. (2022) [19]	USA	Pseudo-randomised and controlled study	48 third-year medical students	Written history and physical (H&P) notes graded with Differential diagnosis, Explanation of reasoning and Alternatives (IDEA) rubric	Addition of clinical reasoning podcasts to the standard curriculum	Grading of weekly H&P notes using the IDEA rubric	No significant improvement in IDEA scores for the podcast intervention group compared to control	IDEA scores from H&P notes	No statistical difference in change of average IDEA scores between intervention and control groups
Bonifacino et al. (2019) [23]	USA	Pseudo-randomised and controlled study	67 third-year medical students	Quiz, IDEA tool, survey on clinical reasoning education	Through six interactive online modules and a skills-based workshop clinical reasoning curriculum	Knowledge quiz, IDEA tool assessment of admission notes, survey on educational experience	Improved knowledge of clinical reasoning concepts, superior utilization of clinical reasoning skills in admission notes	Quiz scores, IDEA tool scores, survey responses	Students in the intervention group demonstrated superior performance on the clinical reasoning knowledge quiz, demonstrated superior written reasoning skills in the data synthesis and diagnostic reasoning portions of their admission notes.
Braun et al. (2019) [20]	Germany	Prospective randomised study	148 fourth- and fifth-year medical students	Electronic learning platform (CASUS) assessments	Problem representation and structured scaffolding for reflection with or without feedback in virtual patient cases	Diagnostic accuracy and efficiency, and analysis of diagnostic errors	Neither structured reflections nor representation scaffolding improved diagnostic accuracy or efficiency compared to control group	Diagnostic accuracy, diagnostic efficiency, and error distribution	Diagnostic accuracy and efficiency did not differ significantly between any groups in the two different assessment phases. The most important causes for diagnostic errors were lack of diagnostic skills, lack of knowledge, and

									premature closure.
Brich et al. (2017) [24]	Germany	Randomised crossover design	122 third- and fourth-year medical students	Multiple-choice question examination (MCQE), key feature problem examination (KFPE)	sTBL sessions covering neuroanatomical localisation, interactive small group seminars	MCQE for knowledge, KFPE for clinical reasoning	No differences in MCQE results between groups; sTBL led to significantly better performance in KFPE for the topic "acute altered mental status"	Performance in MCQE and KFPE, student feedback	sTBL led to significantly better student performance in KFPE after sTBL instruction on "acute altered mental status" but no differences in other topics
Capaldi et al. (2015) [25]	USA	Prospective randomised crossover trial	36 second-year medical students from a single institution	CIP completion during sessions, feedback from faculty developers	Introduction and implementation of the Clinical Integrative Puzzle (CIP) in small group settings	Odd–even item reliability (split-half procedure) for grid questions within each CIP, feedback from participants	High feasibility and acceptable reliability with CIP completion, modest evidence for validity	Feasibility measured by time for CIP completion, reliability measured by odd–even item reliability, validity measured through feedback and performance correlation	Odd–even reliability ranged between 0.43 and 0.73, with a mean of 0.60; significant small to moderate correlations with small group performance
Chadha et al. (2021) [26]	USA	Quasi-experiment al study	94 First-year medical students	Knowledge pre- and post-tests, optional session postsurvey.	Through a virtual workshop emphasising skills in clinical reasoning, differential diagnosis generation, and determination of focused physical exam.	Knowledge gains assessed through pre- and post-tests, postsurvey feedback.	Significant knowledge gains from pre-test to post-test; overall positive student feedback.	Test scores, survey responses.	There was improvement in average scores from 57% to 70% on the five-question test.

Chamberland et al. (2015) [27]	Canada	Quasi-experimental study	53 third-year medical students	Assessment	Through self-explanation while solving clinical cases and then listening to SE examples provided by peers or experts.	Diagnostic accuracy and performance assessed before and after intervention, and one week later on both training and transfer cases.	No significant differences were found between groups in improving diagnostic accuracy or performance on transfer cases; however, all groups improved over time indicating the beneficial effect of self-explanation alone.	Diagnostic accuracy and diagnostic performance scores.	There was improvement in diagnostic performance over time for all groups; no significant differences between groups on transfer cases.
Chamberland et al. (2015) [28]	Netherlands	Quasi-experimental study	54 third year medical students	Diagnostic performance assessment on clinical cases	Combining self-explanation with examples of residents' self-explanations and prompts	Diagnostic performance and accuracy on clinical cases	Group with self-explanations, examples, and prompts showed significantly higher diagnostic performance compared to control	Diagnostic performance and accuracy	Group with prompts showed significant improvement in diagnostic performance compared to control group
Cheng and Senathirajah (2022) [29]	USA	Randomised controlled study	15 third- and fourth-year medical students	Diagnostic tests using clinical data visualisations	Exposure to patient cases through a novel electronic health record briefly and repeatedly (Group A) or twice over a longer period (Group B)	Diagnostic accuracy after exposure to clinical data visualisations	Higher accuracy in diagnostic reasoning in Group A compared to Group B	Diagnostic accuracy	Group A had higher mean percentage correct diagnoses compared to Group B
Chew et al. (2016) [30]	Malaysia	Quasi-experimental study	40 final-year medical students	Assessment based on five case scenarios	Through a 90-minute tutorial on cognitive biases, debiasing strategies, and the TWED (Threat, What else, Evidence and Dispositional factors) checklist	Clinical decision-making assessment based on five case scenarios	Significantly higher scores in the intervention group compared to the control group	Scores on clinical decision-making assessment	The intervention group had significantly higher mean scores compared to the control group

Choi et al. (2020) [31]	South Korea	Randomised controlled trial	87 fourth-year medical students	Pre- and post-rotation tests	Through traditional dermatology electives enhanced with additional educational interventions: 2-h training with reflection and feedback (experimental); 1-h lecture and 1-h outpatient clinic (lecture); and 2-h outpatient clinic (no intervention).	Diagnostic accuracy measured using written clinical cases before and after the educational interventions	Practice with reflection and immediate feedback improved diagnostic accuracy more effectively than the lecture-based approach or the elective alone	Diagnostic accuracy in evaluating dermatologic conditions before and after the interventions	Students in the experimental group showed a significant improvement in diagnostic accuracy, particularly for the conditions included in the training set.
Costa Filho et al. (2019) [32]	Brazil	Quasi-experiment al study	61 sixth-year medical students	Dermatological images, initial and final diagnoses and confidence ratings, structured reflection procedure for the reflection group (RG).	Deliberate reflection procedure on dermatological cases for RG students.	By comparing initial and final diagnostic accuracy and confidence, and calculating calibration.	Reflection increased diagnostic accuracy but did not significantly affect confidence or calibration.	Diagnostic accuracy, confidence, and calibration (alignment between confidence and accuracy).	Reflection group showed increased diagnostic accuracy (49.7% vs 38.4%) compared to control group (CG), but confidence and calibration scores did not differ.
Diemers et al. (2015) [33]	Netherlands	Quasi-experiment al study	13 third-year medical students	Recordings of think-aloud while diagnosing	Through real patient contacts in a PBL curriculum, supported by other educational activities like lectures and laboratory work.	Diagnostic accuracy, case-processing time, use of biomedical and clinical knowledge during diagnostic reasoning, quality of pathophysiological explanations.	Improved diagnostic reasoning skills, developed knowledge networks; however, transfer of knowledge to new problem contexts remained challenging.	Improvements in diagnostic reasoning skills and the quality of knowledge networks; measured changes pre- and post-course.	There was increase in diagnostic accuracy and improvement in pathophysiological explanations; differences were less pronounced for transfer cases compared to course cases.

Findyartini et al. (2016) [34]	Australia and Indonesia	Comparative case study	Medical students and teachers	Diagnostic Thinking Inventory, interviews, focus group discussions	Through PBL and clinical practice, influenced by cultural perspectives	Diagnostic Thinking Inventory scores, qualitative analysis of interviews	N/A	Diagnostic Thinking Inventory (DTI) scores	Students from Universitas Indonesia were found to score lower on the Flexibility in Thinking subscale of the DTI
Fukuta and Morgan (2018) [35]	UK	Randomised controlled trial	40 final-year medical students	Time to investigation and treatment decisions, Ottawa Crisis Resource Management (OCRM) score, questionnaire	Through the step-wise management of a patient presented in a video from a first-person perspective	Measured by 'time to' investigation and treatment decisions and non-technical skills using the OCRM score	Students who watched the video appeared to perform better in clinical decision-making and non-technical skills.	Clinical decision-making performance, non-technical skills, student feedback on the video	100% of students in the intervention group made the correct diagnosis compared with 70% in the control group; higher percentage of students in the intervention group made the correct decisions in nine out of ten decisions.
Gilkes et al. (2022) [36]	Australia	Prospective longitudinal study	235 second year medical students at the pre-clinical stage and 236 at the end-of-year stage	Questionnaires, OSCE assessments, diagnostic reasoning score sheets	Diagnostic reasoning examiner training package and integration of diagnostic reasoning questions into clinical assessments	Through examiner observations during OSCEs, diagnostic reasoning scores, and analysis of common diagnostic errors	Improved examiner confidence in feedback, improved standardisation of diagnostic reasoning teaching and assessment	Examiner feedback confidence, rates of diagnostic reasoning errors in students, student history station scores	Incorporation of diagnostic reasoning training and feedback tools led to improved teaching and assessment of diagnostic reasoning, and changes in student diagnostic error types over time
Gouzi et al. (2019) [37]	France	Randomised-block study	80 third-year medical students	Questionnaire, feedback forms, assessments	Interactive whiteboard (IWB)-based clinical reasoning learning (CRL) sessions	Feedback from students, comparison with traditional courses, pre and post assessments of test ordering and interpretation	There was better alignment of diagnostic tests with hypotheses and indications, improvement in test ordering and interpretation	Student engagement, satisfaction with the session, understanding of clinical reasoning, appropriateness of test ordering and interpretation	There was improvement in identifying diagnostic tests, interpretability of results, and reduction in unnecessary test ordering in IWB group compared to control

Hakim et al. (2023) [38]	United Kingdom	Descriptive cross-sectional study	109 undergraduate medical students in their second year	Audience response system (Mentimeter), surveys	Through an integrated teaching session on respiratory physiology using real-life cases and audience response system (ARS) for immediate feedback	Students' engagement and responses during the ARS session, feedback collected immediately after the session	High levels of student engagement and improved understanding of clinical reasoning and respiratory physiology	Student engagement during the session, understanding of clinical reasoning	There was high consistency in student engagement with ARS throughout the session, majority correctly identified clinical decisions
Harendza et al. (2017) [39]	Germany	Quasi-experimental study	128 final year medical students, 42 complete data sets were available	Self-assessment questionnaire, pre and post course paper case assessments	Through a structured course with learning units on data acquisition, problem presentation, deductive reasoning, pattern recognition, cognitive errors, and dealing with uncertainty	Self-assessment questionnaires, written problem presentations, and differential diagnosis lists before and after the course	Improved self-assessed clinical reasoning skills, more focused case presentations	Self-assessment of clinical reasoning skills, structure and focus of case presentations, number of differential diagnoses	The students assessed themselves significantly better in all eight skills post-course than pre-course. The greatest improvement was in the skill to assess typical reasoning errors and identify situations when they can occur.
Hayward et al. (2016) [40]	Canada	Quasi-experimental study	301 undergraduate medical students at the end of the second preclinical year	Interactive slide bars for likelihood estimates, student feedback	Through an online virtual patient survey system with simulations of history taking, physical examination, laboratory test ordering and reviewing clinical materials in an electronic medical record	Through student responses to the virtual patient case, compared to data from expert clinicians and peers	Students found the case novel, innovative, and clinically authentic; it provided a valuable learning experience	Student diagnostic likelihood estimates, student feedback	Student diagnostic likelihood estimates were consistent year to year but different from expert clinician estimates
Hege et al. (2018) [41]	Europe	Comparative cross-sectional study	317 undergraduate medical students	Concept mapping tool	Use of a concept mapping tool in virtual patient scenarios	Analysis of completed concept maps	Diagnostic accuracy was not a sole indicator for clinical reasoning competency	Differences in clinical reasoning processes based on diagnostic attempts	Authors found significant differences between maps with a correct final diagnosis on one or multiple attempts and maps in which learners gave up and requested

									the solution from the system. These maps had lower scores, fewer summary statements, and fewer problems, differential diagnoses, tests and treatments.
Houchens et al. (2017) [42]	USA	Exploratory qualitative study	12 clinician-educators, 57 current learners, and 26 former learners	Interviews, focus group discussions, direct observations of clinical teaching	Through direct clinical teaching during rounds, leveraging various educational techniques	Based on reflections from all team members including educators and learners, through interviews and observations	Techniques and behaviours of educators identified through observations were supported by reflections from the entire team	Learner reflections, educator behaviours and techniques, improvement in clinical reasoning skills	The techniques and behaviours of educators were categorised into 4 themes 1) emphasising organisation and prioritisation, 2) accessing prior knowledge, 3) thinking aloud, and 4) analysing the literature.
Keemink et al. (2018) [43]	Netherlands	Within-subject experiment al design	32 second-year medical students	Illness script experiment, examination results	Through a structured case-based clinical reasoning (CBCR) course consisting of nine sessions in small groups, covering all stages of clinical encounter	CBCR course examination consisting of cases with multiple questions related to each case	Richness of illness scripts for CBCR diseases was higher compared to non-CBCR diseases; better diagnostic performance on CBCR cases	Illness script richness, diagnostic performance	Significant difference in illness script richness and diagnostic performance between CBCR and non-CBCR diseases
Kelekar and Afonso (2020) [44]	USA	Quasi-experiment al study	198 Second year medical students	Objective structured clinical examination (OSCE)	Through whole case approach, serial cue approach and self-explanation of pathophysiological mechanisms	Performance in OSCE	Students achieved higher scores on differential diagnosis	Scores on differential diagnosis in OSCE	There were higher scores on differential diagnosis for students in the study cohort
Kiesewetter et al. (2016) [21]	Germany	Exploratory cross-sectional study	21 third, fourth- and fifth-year medical students	Transcripts from audio-recorded think-aloud sessions, pre-study questionnaire, knowledge test	Through whole case approach, serial cue approach and self-explanation of pathophysiological mechanisms	Analysis of think-aloud transcripts coded according to diagnostic knowledge categories	The use of diagnostic knowledge categories was not directly related to diagnostic accuracy.	Frequency and time of application of different diagnostic knowledge categories,	Conceptual and strategic knowledge were used more frequently than conditional knowledge. Metacognition correlated with prior knowledge and conceptual knowledge

								diagnostic accuracy	use.
Kiesewetter et al. (2020) [45]	Germany	2x2 factorial design	142 medical students from third to sixth year	Conceptual knowledge pre-test, strategic knowledge pre- and post-tests, final diagnosis accuracy, cognitive load measurement	Use of virtual patients in clinical reasoning training	By measuring changes in strategic knowledge, diagnostic accuracy	No significant difference between case formats in improving strategic knowledge or diagnostic accuracy was observed; prior knowledge level had a significant effect on cognitive load.	Strategic knowledge gain, diagnostic accuracy, intrinsic and extraneous cognitive load	No significant effect of case format on learning outcomes; high prior knowledge was associated with better outcomes, while low prior knowledge was associated with higher cognitive load.
Klein et al. (2019) [46]	Germany	1x3-factorial experiment al design	98 medical students	Web-based learning environment CASUS, pre- and post-tests, cognitive load and self-efficacy assessments	Through clinical case vignettes presenting typical diagnostic errors with additional instructional support for error analysis (unsupported examples, closed prompts, open prompts)	Pre- and post-tests measuring conceptual, strategic, and conditional knowledge; cognitive load; and self-efficacy assessments	Learning from errors approach was effective; however, additional prompting did not enhance learning outcomes.	Improvements in clinical reasoning performance, changes in cognitive load and self-efficacy	There was improvement in clinical reasoning performance from pre- to post-test; neither prompting procedure improved learning outcomes beyond level of unsupported worked example condition.
Kleinert et al. (2015) [47]	Germany	Quasi-experiment al study	62 third-year medical students	Assessments	Teaching module for oesophageal cancer, involving diagnostic and therapeutic decision-making based on standard operating procedures.	Based on correct diagnostic and therapeutic choices in simulator cases and improvements in pre- and post-simulation assessments.	There were significant improvements in clinical reasoning and declarative knowledge post-simulation.	Improvement in clinical reasoning, correct diagnosis and therapy choices, declarative knowledge gains	Significant improvements in diagnosis accuracy (65% to 92%) and correct therapy choice (32% to 76%) post-simulation, increase in correct answers from 5/10 to 7/10 in MCQs.

Koenemann et al. (2020) [48]	Germany	Phenomeno logical study	209 Medical students in various clinical years	Questionnaire	Through structured approach working on complex medical cases in clinical case discussions (CCD) format.	Self-assessment of clinical reasoning competence using an established clinical reasoning questionnaire at the beginning and end of CCD courses.	High acceptance and significant improvements in clinical reasoning self-assessments among participants.	Self-assessment clinical reasoning scores; participant feedback	Significant increases in overall clinical reasoning scores from introduction to exit evaluation.
Lai et al. (2022) [49]	Taiwan	Quasi-experiment al study	76 Fourth-year medical students, five teachers, and five standardised patients	scoring trainee's group history taking and post-study questionnaires.	Group history-taking with individual reasoning principles with five standardised patients covering different clinical scenarios, and immediate feedback from instructors	Assessment based on students' history taking, documentation of key information, and ability to make correct diagnoses.	Identified areas of difficulty in students' clinical reasoning, improved understanding through immediate feedback.	History-taking scores, key information documented, correct diagnosis rates and post-study questionnaire feedback.	Training improved clinical reasoning skills. There was a correlation in the clinical reasoning process between the correct and incorrect most likely diagnosis groups.
Leung et al. (2015) [50]	Hong Kong	2 x 2 crossover study	130 fifth-year medical students	End-of-module multiple choice questions test, modified essay questions tests and Teaching and Learning Resources Centre login	Virtual patient sessions on acute pain management	Performance in acute pain management items set within exams; analysis of student login times	Formative assessment case studies enhanced performance in all examinations; storyline virtual patient had no demonstrable effect.	Student performance in exams, student teaching evaluation questionnaires, student login times	Formative assessment case studies reinforced learning, whereas the storyline virtual patient's educational role was less clear.
Lütgendorf-Caucig et al. (2017) [51]	Austria	Phenomeno logical study	Undergraduat e medical students interested in clinical oncology, basic research and international contacts.	Pre-VSSO and post-VSSO student evaluation	Case-based exercises, clinical reasoning seminars, bedside teaching methods, interactive patient cases at the Vienna Summer School on Oncology (VSSO) program	Pre- and post-course knowledge checks	There was significant knowledge acquisition in general and specific aspects of cancer.	Improvement in students' knowledge about cancer and clinical reasoning in a multidisciplinary setting.	Student evaluation at the beginning and end of the program indicated significant knowledge acquisition in general and specific aspects of cancer.

Maciuba et al. (2023) [52]	USA	Longitudinal qualitative study	70 First- and second-year medical students	Video recordings of small-group sessions, thematic analysis of transcripts	Introduction to Clinical Reasoning course focussing on case-based learning emphasising diagnostic reasoning, using prototypical examples of diseases applied to case-based learning	Analysis of teaching sessions transcripts for themes associated with clinical reasoning processes and knowledge organisation	Preceptors emphasised problem lists, differential diagnoses, and leading diagnoses, used illness scripts implicitly rather than explicitly	Thematic sufficiency in transcripts analysis, prevalence and explicitness of clinical reasoning themes	Preceptors in pre-clerkship clinical reasoning course focused on problem list creation, differential diagnoses, and context of care.
Middeke et al. (2018) [53]	Germany	Prospective study	112 final-year medical students	Formative examination consisting of six key feature cases and a final 45-minute Serious Game, (EMERGE) session	Participation in ten 90-minute teaching sessions of either EMERGE or PBL	Formative key feature examination and final EMERGE session	The EMERGE group scored significantly higher than the PBL group in the key feature examination	Performance in key feature examination and final EMERGE session	EMERGE group scored significantly higher in key feature examination; In the final EMERGE session, the EMERGE group achieved significantly better results than the PBL group.
Middeke et al. (2020) [54]	Germany	Prospective randomised study	69 fifth-year medical students	EMERGE log files, performance scores in Serious Game sessions	Through a computer-based emergency ward simulation game (Serious Game) involving up to 46 different virtual patient cases	Based on performance scores and activities within the Serious Game	Learning with Serious Games facilitates the acquisition of higher-order cognitive functions which may transfer to managing virtual patients with similar complaints.	Student performance in the final Serious Game session, transfer of clinical reasoning skills	Exposed students scored significantly higher than unexposed students in the cases 'NSTEMI' and 'asthma exacerbation'; no significant differences in other cases.
Moghadami et al. (2021) [55]	Iran	Randomised control trial	100 fourth-year medical students	Internally developed knowledge test and a Script Concordance Test (SCT).	Teaching workshops using illness script method	Post-test scores on internally knowledge test and SCT	Post-test scores in the intervention group were significantly higher than the control group. Learner satisfaction data indicated that the	Differences in post-test scores	The illness script method improved students' clinical reasoning skills

							intervention was well-received by students.		
Mutter et al. (2020) [56]	USA	Randomised controlled trial	96 Fourth-year medical students	64-question script concordance test (SCT)	Patient case scenarios with a manikin or without a manikin for chest pain sessions.	Clinical reasoning skills were assessed post-session using a script concordance test.	Use of a manikin in simulated patient case discussion significantly improved students' clinical reasoning skills as measured by SCT scores.	Differences in SCT scores between students in manikin versus non-manikin groups.	Statistically significant mean difference in SCT scores between the two groups, with the manikin group achieving higher scores.
Peacock and Grande (2015) [57]	USA	Repeated measures design	47 first-year pathology students	Differential diagnosis assignment, student survey	Students interacted with real patients presenting their histories without revealing their diagnoses, then formulated and submitted differential diagnoses.	Performance in formulating differential diagnoses based on real patient histories, clinical reasoning skills through online assignments	There was improved clinical decision-making skills, integrated basic sciences with patient's clinical presentation and improved empathy towards patient experiences.	Improvement in differential diagnosis formulation, student confidence in clinical decision-making, understanding of patient challenges.	Real patient exposure improved students' clinical decision-making skills and helped to provide clinical context to the basic sciences they were learning.
Plackett et al. (2020) [58]	UK	Randomised controlled trial	264 final year undergraduate students	Survey, Self-reported Flexibility in Thinking (FIT) scale of the Diagnostic Thinking Inventory (DTI), additional eCREST patient case, multiple choice questions.	eCREST, an online simulation tool, showing three videos of patients presenting to their primary care physician with respiratory problems	Measured using the FIT scale of the DTI and observed measures of clinical reasoning using data from an additional eCREST patient case	Improved data gathering skills that could reduce diagnostic errors, was highly acceptable to students	Uptake and retention rates, student feedback on eCREST, scores on FIT scale and observed measures of clinical reasoning	eCREST improved students' ability to gather essential information from patients

Raupach et al. (2021) [59]	Germany	Prospective cohort trial	178 students enrolled in either the fourth or the fifth year of undergraduate medical education	Log-files of in-game activity	Through a serious game simulating an accident and emergency department where students manage virtual patients over six 90-min sessions	Analysis of log-files of in-game activity regarding history taking and patient management in three virtual patient cases.	Overall performance scores increased significantly from the beginning to the end of the training phase, and performance remained stable over 1.5 years.	Overall performance scores of changes in history taking and patient management scores.	Performance scores increased from 57.6% to 65.5% during the training phase, and ever-exposed students showed better management scores than non-exposed students (72.6% vs 63.5%).
Roberti et al. (2016) [60]	Brazil	Cross-sectional exploratory descriptive study	40 First to Fifth year medical students from first to fifth year	Focus group discussions	Through traditional medical education curriculum with division into preclinical and clinical phases	Through content analysis of focus group discussions	The was change in clinical reasoning from knowledge-based in the preclinical phase to pattern recognition in the clinical phase	Student statements and discussions in focus groups about clinical reasoning and knowledge of basic medical science	93.1% of preclinical phase students based their clinical reasoning on basic medical science, while in the clinical phase, students progressively moved to pattern recognition
Rosby et al. (2018) [61]	Singapore	Within-subjects incomplete block design	32 second-year medical students	Qualtrics survey tool	Participants were shown eight online chest X-ray cases during a training phase; half were trained repeatedly, half were shown only twice.	Diagnostic accuracy and response time in a final test phase where all eight cases were shown again	Training resulted in significant improvements in diagnostic accuracy and reduced response time for trained cases	Diagnostic accuracy (out of 4) and response time (seconds)	There were significantly higher diagnostic accuracy and faster response time for trained cases compared to untrained.
Rumayyan et al. (2018) [62]	Saudi Arabia	Randomised controlled study	88 second-year medical students	Diagnostic performance assessment on clinical cases	Instructional approach (self-explanation vs. hypothetico-deduction) in diagnosing clinical cases. Under self-explanation condition, students provided a diagnosis	Mean diagnostic accuracy score on follow-up test involving similar cases	Students in the hypothetico-deduction condition outperformed those in the self-explanation condition	Mean diagnostic accuracy score	Mean diagnostic accuracy was higher in the hypothetico-deduction condition compared to the self-explanation condition

					and pathophysiological explanation for clinical findings. In hypothetico-deduction condition, students hypothesised about diagnoses for sequentially presented signs and symptoms.				
Schubach et al. (2017) [22]	Germany	Quasi-randomised controlled trial	56 fourth- and fifth-year medical students	Script concordance test	Use of virtual patients with different instructional approaches	Script concordance test (SCT) measuring students' clinical reasoning capacity	No significant improvement in SCT scores for the podcast intervention group compared to control	SCT scores	No significant difference in SCT scores between the key feature and systematic arms.
Schuelper et al. (2019) [63]	Germany	Monocentric, prospective nonrandomised study	75 fourth-year medical students	Formative key feature examinations	Through weekly computer-based seminars using patient case histories containing key feature questions, available in text and video formats, with students choosing their preferred presentation format at the beginning of each case seminar.	Performance in key feature questions assessed in formative entry, exit and retention exams	Students with higher exposure to video-based items scored higher in the retention exam compared to those with higher exposure to text-based items, despite the overall preference for text-based case histories.	Performance in key feature questions during entry, exit and retention exams	No significant difference in entry and exit exams between groups. Significant difference in retention exam scores (75.3% in video-preference vs 63.4% in text-preference group) was recorded.
Scott et al. (2020) [64]	UK	Quasi-experimental study	45 final-year medical students	Debriefs facilitated by researchers using the 'Diamond	Through a simulation teaching day including 'uncertainty' simulation replacing	Structured debriefing following simulations, using advocacy and inquiry to help students reflect	Students found 'uncertainty' simulation more challenging, disheartening when	Student reflections during debriefings, changes in perception	Simulation encouraged expectations of textbook diseases, leading to challenges in adapting to complex patient

				Approach', transcribed and analysed thematically	one of six existing scenarios		no clear diagnosis was reached, struggled with task fixation/inaction and identifying when to seek senior support	towards clinical uncertainty	encounters. Students struggled to act without a clear diagnosis and were unsure of their role in such situations.
Weidenbusch et al. (2019) [65]	Germany	Single-centre randomised controlled trial with parallel design	90 Undergraduate medical students (level not specified)	Knowledge application test	Live discussion (Live-CCD), watching recordings of the live discussions (Video-CCD) or working with printed cases (Paper-Cases).	Pre-test, post-test, and delayed post-test using a knowledge application test	Live-CCD group displayed the highest learning outcomes, followed by Video-CCD and Paper-Cases. No difference was found between Live-CCD and Video-CCD groups in the delayed post-test; however, both outperformed the Paper-Cases group	Improvements in clinical reasoning skills as measured by the knowledge application test. Questionnaire for subjective learning outcomes.	Significant improvements in knowledge application post-test scores for Live-CCD and Video-CCD groups compared to Paper-Cases. No significant difference between Live-CCD and Video-CCD in delayed post-test.
Wu et al. (2014) [66]	China	Quasi-experimental study	29 Year 3 to Year 5 medical students	Pre-test and post-test, assessment of learning products (dual maps generated by learners), survey	Four-week online learning program with computer-based cognitive representation for clinical reasoning	Knowledge tests (pre-test and post-test), evaluation of learners' dual maps, survey on perceived learning gains	No significant difference between pre-test and post-test scores, but there was significant improvement in learning products (dual maps) from the beginning to the end of the study	Test scores, dual map scores, survey responses	Significant improvement in overall performance in dual maps, improvement in connections from problem-solving to knowledge construction. No significant differences were found between the pre-test and post-test scores with the 4-week period

Wu et al. (2016) [67]	Hong Kong	Quasi-experiment al study	52 third-year or higher students	Cognitive maps and verbal text, pre-test and post-test	Simulated cases of kidney disease in an online system, with either cognitive-mapping or verbal-text approach, focusing on data capture, hypotheses formulation, reasoning with justifications, concept identification and concept relationships.	Based on the reasoning process (data capture, hypotheses formulation, reasoning with justifications) and the construction of knowledge (concept identification, concept relationships).	There was improvement in problem-solving performance and reasoning process for cognitive-mapping group, but no significant differences in subject-matter knowledge test between groups.	Problem-solving performance, subject-matter knowledge test scores	Cognitive-mapping approach improved reasoning process but not knowledge construction compared to verbal-text approach. No significant differences in subject-matter knowledge test scores between groups.
Zagury-Orly et al. (2022) [68]	USA	Quasi-experiment al study	331 first-year medical and dental students	Online survey, academic quiz performance, team discussions	Introduction and implementation of the Student-Generated Reasoning Tool (SGRT) in small group settings, whereby students proposed and justified pathophysiological hypotheses, generated new findings and critically appraised information.	Through academic quiz performance, effectiveness of collaborative environments using SGRT, and student feedback	Students were five times more likely to get questions correct in the SGRT group versus control group. Accuracy of pathophysiological hypotheses was significantly lower for individuals than teams.	Academic quiz performance, individual vs. team responses, coding of team discussions, student feedback	Students in the SGRT group had significantly higher scores on relevant quiz questions compared to control group. Team responses were more accurate than individual responses.
